# Salivary microRNA Expression and Co-Expression Patterns in Periodontal Disease: A Cross-Sectional Analysis of miR-34a, miR-155 and miR-146a

**DOI:** 10.3390/cells15151392

**Published:** 2026-07-31

**Authors:** Elena-Teodora Tâlvan, Liviuța Budișan, Călin Ilie Mohor, Valentin Grecu, Ioana Berindan-Neagoe, Cornelia Braicu, George Călin Oprinca, Cosmin-Ioan Mohor, Adrian Nicolae Cristian

**Affiliations:** 1 Faculty of Medicine, “Lucian Blaga” University of Sibiu, 550169 Sibiu, Romania; elena.talvan@ulbsibiu.ro (E.-T.T.); georgecalin.oprinca@ulbsibiu.ro (G.C.O.); cosmin.mohor@ulbsibiu.ro (C.-I.M.); adrian.cristian@ulbsibiu.ro (A.N.C.); 2 Department of Genomics, MEDFUTURE Institute for Biomedical Research, Iuliu Hațieganu University of Medicine and Pharmacy Cluj-Napoca, 400012 Cluj-Napoca, Romania; liviuta.petrisor@umfcluj.ro (L.B.); ioananeagoe29@gmail.com (I.B.-N.); braicucornelia@yahoo.com (C.B.); 3 Faculty of Engineering, “Lucian Blaga” University of Sibiu, 550025 Sibiu, Romania; valentin.grecu@ulbsibiu.ro

**Keywords:** periodontal disease, microRNA, saliva, biomarker, qRT-PCR, clinical attachment loss, miRNA co-expression, confounding, precision dentistry

## Abstract

**Highlights:**

**What are the main findings?**

- Individual salivary expressions of miR-34a, miR-155, and miR-146a were not sufficient to discriminate periodontal disease from health in this cohort.
- Patient age, rather than any single miRNA, was the strongest discriminator between groups, identifying age as a key confounder in salivary miRNA biomarker studies.

**What are the implications of the main findings?**

- Future salivary biomarker studies in periodontology should prioritize integrated multi-miRNA and regulatory network approaches, rather than relying on single-miRNA expression levels.
- Accounting for biological confounders, particularly age, will be essential for developing robust and clinically applicable salivary miRNA biomarkers for periodontal disease.

**Abstract:**

MicroRNAs (miRNAs) have been proposed as promising biomarkers for periodontal disease because of their role in regulating inflammation and tissue remodeling. This study evaluated the salivary expressions of miR-34a, miR-155, and miR-146a and their relationships with periodontal disease and its clinical severity. Saliva samples were collected from 35 patients with periodontal disease and 35 healthy controls, and miRNA expression was quantified by quantitative real-time PCR (qRT-PCR). Fold-change values were log2-transformed, and analyses included differential expression (Mann–Whitney U with Benjamini–Hochberg correction), Spearman correlation with clinical attachment loss (CAL), logistic regression/ROC modeling with internal cross-validation, and an exploratory differential co-expression analysis. None of the three miRNAs was significantly differentially expressed between groups (all FDR-adjusted *p* > 0.40; the result was unchanged after adjustment for age), and none correlated with CAL after accounting for the healthy-versus-diseased separation. miR-34a showed poor stand-alone diagnostic performance (AUC = 0.58; five-fold cross-validation 0.56). Patients were significantly older than controls (41.1 vs. 37.1 years; *p* < 0.001), and age was the strongest single discriminator (AUC = 0.82), indicating that age acts as an important confounder. In an exploratory analysis, the positive co-expression observed among the three miRNAs in healthy controls was attenuated in disease (e.g., miR-155/miR-146a Spearman rho = 0.68 vs. 0.27; permutation *p* approximately 0.06). These findings indicate that salivary miR-34a, miR-155 and miR-146a abundance do not discriminate periodontitis in this cohort, but underline age as a critical confounder and suggest that altered miRNA co-expression, rather than differential abundance, may warrant further investigation in larger, age-matched studies. In addition, miR-34a and miR-146a showed reduced expression dispersion in disease; because the reduced dispersion and the reduced co-expression cannot be fully separated in a sample of this size, these network-level observations are regarded as jointly hypothesis-generating.

## 1. Introduction

Periodontal disease remains a significant global public health issue, affecting billions worldwide. Studies indicate that the global burden of periodontal disease has substantially increased from 1990 to 2019, with over 1 billion cases recorded in 2019. Periodontal disease incidence remains a widespread health concern globally, with an estimated 1.09 billion existing cases and around 91.5 million new cases diagnosed each year [1]. Severe periodontitis affects approximately 11% of the global population, making it the sixth most prevalent condition globally. In the United States and Europe alone, severe periodontitis affects approximately 10–11% of the adult population [2]. The incidence and burden of periodontal disease in Europe remain substantial despite advancements in dental care. Recent estimates suggest that about 10% of the European population is affected by severe forms of periodontal disease, making it a significant public health concern. The economic impact is also considerable, with direct and indirect costs related to periodontal disease reaching approximately €158.64 billion across Europe in 2018, largely driven by productivity losses and edentulism [3]. The burden is even higher in developing regions such as Western Sub-Saharan Africa and parts of Asia, where socioeconomic disparities contribute to increased disease prevalence and severity [4,5]. Age and gender play important roles in the incidence and severity of periodontal disease. Studies show that periodontal disease becomes significantly more prevalent and severe with increasing age, with older adults and seniors displaying higher rates of periodontitis compared to younger groups [6,7]. Research also indicates that, while both genders are affected, men generally show worse periodontal outcomes, such as greater pocket depth and attachment loss, although some biochemical markers of inflammation are higher in women [8]. Gender-based microbial differences have also been observed, with males displaying greater diversity and different dominant bacterial species in the subgingival microbiome [9]. Overall, aging significantly increases the risk and progression of periodontal disease, while gender differences affect the type and biological behavior of the disease. Understanding these factors is crucial for developing targeted prevention and treatment strategies.

Biomarkers are important tools across various diseases, offering valuable insights for early diagnosis, prognosis, disease monitoring, and therapeutic response evaluation. In conditions such as cancers, cardiovascular diseases, autoimmune disorders, and infectious diseases, they enable precise disease characterization and help tailor personalized treatment approaches [10]. More recently, these tools have become increasingly important in the diagnosis, prognosis, and management of periodontal disease. Traditional clinical methods mainly assess the history of tissue destruction, but fail to indicate current disease activity, making biomarkers a crucial advancement for early detection and monitoring [11,12]. Salivary, gingival crevicular fluid (GCF) and serum biomarkers such as interleukin-1β (IL-1β), tumor necrosis factor-alpha (TNF-α), and matrix metalloproteinase-8 (MMP-8) have shown strong correlations with periodontal disease severity and response to therapy [13].

MicroRNAs (miRNAs) are small, non-coding RNA molecules, approximately 18–25 nucleotides in length, that regulate gene expression post-transcriptionally by binding to complementary sequences on target messenger RNAs (mRNAs), leading to their degradation or translational repression [14,15,16]. While acute upregulation of miR-34a has been associated with cellular senescence and mitochondrial stress pathways in specific epithelial models, its role in the chronic microenvironment of periodontal tissues involves complex homeostatic regulation. Chronic dysregulation or a significant loss of miR-34a expression may lead to the decompression of target oncogenes and pro-inflammatory signaling axes, contributing to unchecked local tissue degradation [17,18,19]. MiR-155 has been identified as a key regulator in the pathogenesis and progression of periodontal disease. Elevated levels of miR-155 have been detected in both saliva and gingival tissues of patients with periodontitis, where its expression correlates with clinical markers of disease severity such as probing depth, gingival index, and attachment loss [20]. MiR-155 contributes to periodontal inflammation by modulating macrophage polarization towards the pro-inflammatory M1 phenotype, especially in patients with comorbidities such as type 2 diabetes, thereby exacerbating tissue destruction [21]. Furthermore, miR-155 levels were shown to positively correlate with elevated concentrations of inflammatory cytokines like TNF-α and IL-6 in patients with chronic periodontitis, suggesting its active role in sustaining chronic inflammation [22]. These findings highlight miR-155 not only as a potential biomarker for periodontal disease severity, but also as a promising therapeutic target to modulate immune responses and improve disease outcomes. MiR-146a is a critical regulator of immune responses and inflammation, playing major roles in the progression of cancer and chronic inflammatory diseases. It suppresses pro-inflammatory pathways like NF-κB, helping maintain immune balance [15,23]. MiR-146a plays a crucial anti-inflammatory role in the pathogenesis and progression of periodontal disease. Studies have shown that miR-146a is significantly upregulated in the saliva and gingival tissues of patients with chronic periodontitis, where its expression correlates with clinical indicators such as probing depth, plaque index, and clinical attachment loss [20]. MiR-146a expression is shown to suppress periodontal inflammation by targeting molecules such as IRAK1 and TRAF6, key players in the Toll-like receptor (TLR) signaling pathway [24]. These findings highlight miR-146a as both a biomarker for disease severity and a promising target for therapeutic strategies aimed at modulating immune responses in periodontitis.

The aim of this study was to investigate the salivary expression patterns of miR-34a, miR-155, and miR-146a in periodontal disease, and to evaluate whether these miRNAs have biomarker potential. Beyond assessing differential expression, we explored miRNA co-expression patterns and their relationships with clinical features to provide a broader perspective on the molecular alterations associated with periodontal disease.

## 2. Materials and Methods

### 2.1. Study Design and Data Collection

The present study included 70 participants, comprising 35 patients with periodontitis and 35 healthy controls. The cohort (n = 70) included both females and males, aged between 35 and 45 years. The two groups had a comparable sex distribution, although patients were, on average, older than controls. Periodontal clinical parameters were evaluated for all patients, primarily recording the clinical attachment loss (CAL) and the presence of bone resorption. The disease severity in the PD group was classified into three stages: incipient, moderate, and advanced.

The study participants were categorized into two groups based on the presence or absence of periodontal disease. Healthy subjects-individuals exhibited good oral hygiene, no clinical signs of gingival attachment loss, no radiographically detectable bone resorption, and no history of periodontal disease or tooth loss attributable to periodontal pathology. Subjects with periodontal disease were those with more than 30% of dental units affected, clinical attachment loss ranging from 2 to 6 mm, accumulation of cervical plaque or tartar covering up to two-thirds of the exposed tooth surfaces, and bone resorption measuring between 2 and 5 mm. Bone resorption was quantified on eight teeth, measured from the cementoenamel junction to the apical junction at a minimum of six sites per tooth, with values averaged arithmetically. Gingival attachment loss was clinically assessed using a periodontal probe at six sites per hemiarch. All clinical and radiographic measurements were performed by a single calibrated examiner to ensure consistency.

This study was approved by the Ethics Committee of Iuliu Hațieganu University of Medicine and Pharmacy in Cluj-Napoca, approval no. 140, from 2 April 2018.

### 2.2. Sample Collection

Unstimulated saliva samples were collected between 9:00 and 12:00 p.m. (noon). Participants were instructed to refrain from eating, drinking, or chewing for at least one hour prior to collection. Before sampling, subjects rinsed their mouths with water and waited at least ten minutes. Saliva was collected over approximately 10 min into sterile Eppendorf tubes by asking participants to first swallow and then lean forward and allow saliva to pool and expectorate. Samples were frozen at −40 °C immediately upon collection and transferred to −80 °C for long-term storage, with no more than one freeze–thaw cycle permitted. Samples contaminated with blood or improperly stored were excluded from analysis. Genetic analysis of the biological material was conducted at the Department of Genomics, MEDFUTURE Institute for Biomedical Research, Iuliu Hațieganu University of Medicine and Pharmacy, Cluj-Napoca. The cohort was stratified into four groups: healthy controls, early periodontitis group, moderate periodontitis group, and advanced periodontitis group. Before enrollment, all participants received a detailed explanation of the study objectives and provided written informed consent after undergoing a comprehensive dental evaluation. Inclusion criteria for the periodontal disease group followed the case definition given in Section 2.1 (clinical attachment loss of 2–6 mm affecting more than 30% of dental units, with radiographic bone resorption of 2–5 mm), together with bleeding on probing, the presence of gingival or bone pockets, and a dental radiograph taken within the previous six months. Exclusion criteria included periodontal treatment within the previous 12 months, smoking, pregnancy, cardiovascular, respiratory, or renal diseases, diabetes, osteoporosis, rheumatoid arthritis, viral, infectious, or autoimmune diseases, cancer, and the use of antibiotics or other systemic treatments within the past six months.

### 2.3. RNA Isolation and qRT-PCR

Total RNA was extracted from saliva samples using TriReagent (Invitrogen, Wilmington, DE, USA), following the manufacturer’s recommended protocol. RNA concentration and quality were evaluated with a NanoDrop-1000 spectrophotometer (Thermo Fisher Scientific, Wilmington, DE, USA) by measuring UV–Visible light absorbance, and sample purity was determined through spectral data and purity ratios. For miRNA expression analysis, a fixed total input of 100 ng of total RNA was reverse transcribed into complementary DNA (cDNA) using the TaqMan MicroRNA Reverse Transcription Kit (Applied Biosystems, Waltham, MA, USA). MiRNA amplification was carried out using the TaqMan Fast Advanced Master Mix (Applied Biosystems). Quantitative real-time PCR (qRT-PCR) was performed on a ViiA 7 System in a 5 µL reaction volume, utilizing a 384-well plate format with a cDNA dilution of 1:5.

Table 1 summarizes the endogenous controls (RNU48 and U6) and the target miRNAs (miR-34a, miR-146a, and miR-155) included in the qRT-PCR analysis, together with their nucleotide sequences and TaqMan assay identifiers. Relative miRNA expression was calculated using the comparative 2^(-ΔΔCt) method, normalized to the geometric mean of the endogenous controls RNU48 and U6 and calibrated to the healthy control group. A stability analysis confirmed that the raw Ct values of RNU48 and U6 did not differ significantly between the control and disease groups (*p* > 0.05). A fixed total input of 100 ng of total RNA was reverse-transcribed for each reaction.

### 2.4. Statistical Analysis

Data analysis was performed using the Python programming language (version 3.x), along with dedicated packages for data science and statistics: pandas for data processing, SciPy and statsmodels for statistical testing and modeling, and scikit-learn for multivariate analysis and machine learning. Graphs were generated using the seaborn and matplotlib libraries. The data set used for this paper is available as Supplementary Materials.

The analytical steps included:

1. Data Transformation: the 2^(-ΔΔCt) fold-change values were log2-transformed to normalize the distribution, correct the right-skewness typical of molecular data, and ensure symmetric scaling of up- and down-regulation.
2. Descriptive and Inferential Statistics: Normality of distribution was assessed using the Shapiro–Wilk test. Because the data did not follow a perfectly normal distribution even after transformation, the non-parametric Mann–Whitney U test was used to compare miRNA expression between the PD and CTR groups. For comparisons across multiple severity stages, the Kruskal–Wallis test was applied. Because the two groups differed in age, the between-group comparison was additionally repeated with adjustment for age using logistic regression (group as the outcome, with each miRNA and age as predictors) and analysis of covariance (miRNA as the outcome, with group and age as predictors).
3. Correlations: to evaluate the relationship between continuous/ordinal variables (e.g., log2 expression levels and CAL values), the Spearman’s rank correlation coefficient (ρ) was calculated, as it is appropriate for non-parametric relationships.
4. Differential Co-expression: To test whether the coordination between miRNAs differed between groups, pairwise Spearman correlations of log2 expression were compared between controls and patients using Fisher r-to-z tests and a 20,000-iteration label-permutation test; differences in dispersion were assessed with the Levene and Fligner–Killeen tests. Relative expression was normalized to the geometric mean of the stable endogenous controls RNU48 and U6, following current recommendations for miRNA RT-qPCR normalization [25].

The threshold for statistical significance was set at *p* < 0.05.

## 3. Results

### 3.1. Demographic and Clinical Characteristics

The study cohort included 70 participants equally distributed between the healthy control group and the periodontal disease (PD) group. Patients diagnosed with periodontal disease were significantly older than controls (41.1 ± 3.6 vs. 37.1 ± 2.6 years; Mann–Whitney *p* < 0.001) (see Table 2). Because increasing age is itself a well-established correlate of periodontal tissue destruction [6,26], this age imbalance was treated as a potential confounder in the subsequent analyses (Section 3.4 and Section 4).

The gender distribution was relatively balanced between groups, minimizing the potential influence of sex-related bias on the molecular analyses. Within the PD cohort, the majority of patients exhibited moderate disease severity (n = 24), while smaller proportions presented incipient (n = 5) or advanced forms (n = 6) of periodontitis. This distribution suggests that the analyzed cohort predominantly reflected intermediate stages of periodontal progression, and the incipient (n = 5) and advanced (n = 6) subgroups were too small to support robust stage-specific inference (Section 6).

### 3.2. MiRNA Expression Analysis

Comparison of log2-transformed expression between groups revealed no statistically significant differences for any of the three miRNAs. miR-34a, miR-155 and miR-146a all had similar median expression in patients and controls, and none remained significant after Benjamini–Hochberg correction (miR-34a *p* = 0.28, FDR = 0.42; miR-155 *p* = 0.66, FDR = 0.66; miR-146a *p* = 0.13, FDR = 0.40; Table 3). Thus, in this cohort, none of the analyzed miRNAs discriminated periodontitis from health on the basis of abundance. Because the patients were significantly older than the controls (Section 3.6), this comparison was repeated with adjustment for age: after adjustment, none of the three miRNAs reached statistical significance (age-adjusted *p* = 0.06, 0.29 and 0.06 for miR-34a, miR-155 and miR-146a, respectively), whereas age itself was strongly associated with group membership in every model (*p* < 0.0001). Taking age into consideration did not change the results—there is still no significant difference.

**Figure 1:**
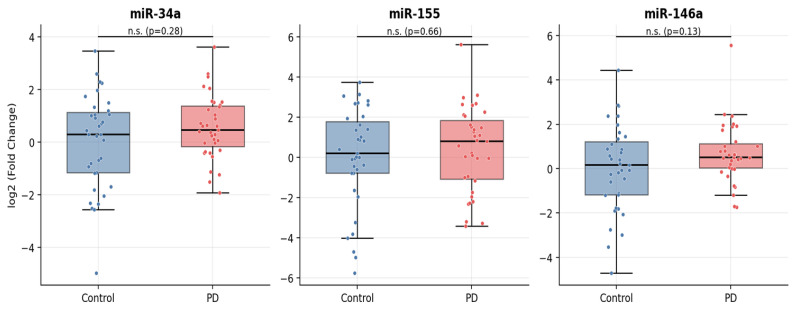
Comparative expression profiles of miR-34a, miR-155, and miR-146a.

The boxplots in Figure 1 show largely overlapping expression distributions for all three miRNAs between healthy controls and periodontal disease patients. No comparison reached statistical significance (Mann–Whitney *p* = 0.28, 0.66 and 0.13 for miR-34a, miR-155 and miR-146a, respectively). Figure 1 therefore illustrates the absence of a differential abundance signal for these salivary miRNAs in the studied cohort.

**Figure 2:**
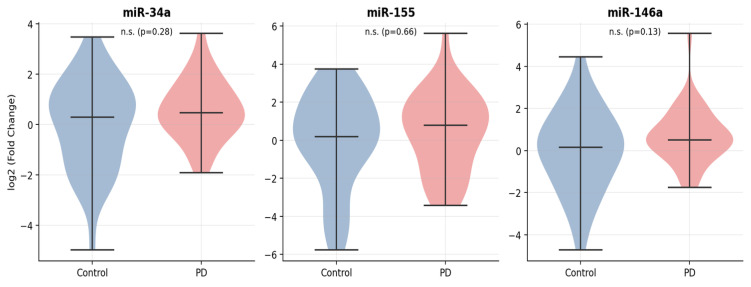
Violin plot analysis of miRNA expression.

Figure 2 illustrates the distribution of log2-transformed expression levels of miR-34a, miR-155, and miR-146a in healthy controls (CTR) and patients with periodontal disease (PD) as violin plots. Consistent with the boxplots, the distributions overlap extensively and no miRNA differs significantly between groups (all *p* > 0.10). The plots also indicate that miR-34a and miR-146a occupy a somewhat narrower range in the disease group, a dispersion difference examined further in Section 3.6.

Overall, miR-34a, miR-155 and miR-146a displayed no group-level shifts in salivary abundance, confirming that, within this study population, none of the three miRNAs behaves as a differentially expressed marker of periodontal disease.

### 3.3. Correlation with Clinical Severity (Clinical Attachment Loss—CAL)

**Figure 3:**
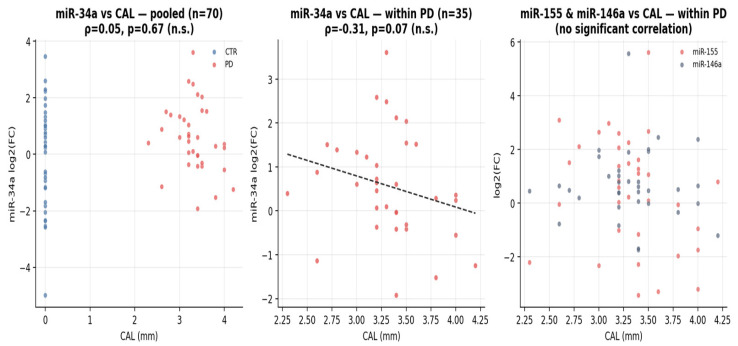
Correlation between miRNA expression and clinical attachment loss (CAL).

To determine whether molecular alterations reflect periodontal tissue degradation, the relationship between log2-transformed miRNA expression levels and clinical attachment loss (CAL), the principal clinical parameter of periodontal disease severity, was evaluated using Spearman correlation analysis (see Figure 3). Across the pooled cohort, no miRNA showed a meaningful correlation with CAL (miR-34a rho = 0.05, *p* = 0.67; miR-155 rho = −0.03, *p* = 0.79; miR-146a rho = 0.16, *p* = 0.18). Because all controls have CAL = 0, the correlation was also tested within the periodontal disease group only (n = 35), where a clinically meaningful severity gradient could exist. Within the PD group, miR-34a and miR-155 showed only weak negative trends with CAL (rho = −0.31, *p* = 0.07 and rho = −0.32, *p* = 0.06, respectively) that did not reach significance and did not survive correction for multiple testing; miR-146a showed no association (rho = −0.02). These weak within-group trends persisted after adjustment for age (partial rho approximately −0.30, *p* approximately 0.07–0.09), but should be regarded as hypothesis-generating only, given the small sample and the number of comparisons performed. No miRNA was significantly correlated with probing depth or radiographic bone resorption after false-discovery-rate correction (all FDR > 0.30).

Overall, these analyses provide no evidence that salivary miR-34a, miR-155 or miR-146a tracks periodontal disease severity as a continuous, dose-dependent marker.

### 3.4. Correlation Analysis of Molecular and Clinical Variables

**Figure 4:**
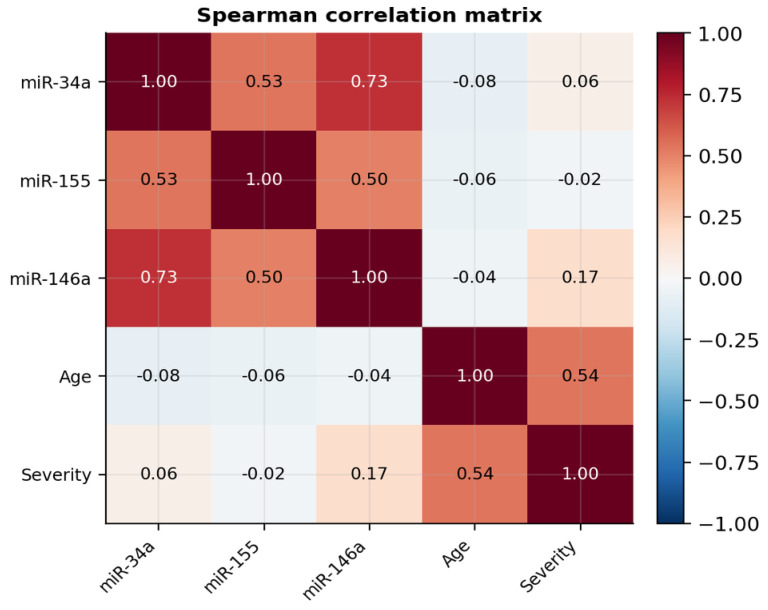
Spearman correlation matrix of molecular and clinical variables.

To explore the interactions between molecular biomarkers and clinical parameters, a Spearman correlation matrix was constructed (Figure 4). The three miRNAs were positively inter-correlated, most strongly between miR-34a and miR-146a (rho = 0.73) and between miR-155 and miR-146a (rho = 0.50, *p* < 0.001), consistent with shared upstream regulation. miR-34a expression was essentially uncorrelated with patient age (rho = −0.09, *p* = 0.48) and with the severity score (rho = 0.06, *p* = 0.61), whereas age was moderately correlated with severity (rho = 0.54, *p* < 0.001). An exploratory comparison of the correlation structure between groups indicated that the coordinated co-expression seen in controls was attenuated in disease (mean |rho| 0.72 vs. 0.47; miR-155/miR-146a rho = 0.68 vs. 0.27; label-permutation *p* approximately 0.06 for the strongest pairs; Figure 4).

This reframes the molecular signal in the cohort as a possible network-level phenomenon: while individual salivary miRNA levels do not distinguish periodontitis, the coordination among these inflammation-associated miRNAs appears to be perturbed in disease [27,28]. Given the borderline significance and the modest sample, this observation is presented as hypothesis-generating and requires validation in larger, age-matched cohorts.

### 3.5. Differential Co-Expression and Dispersion Analysis

**Figure 5:**
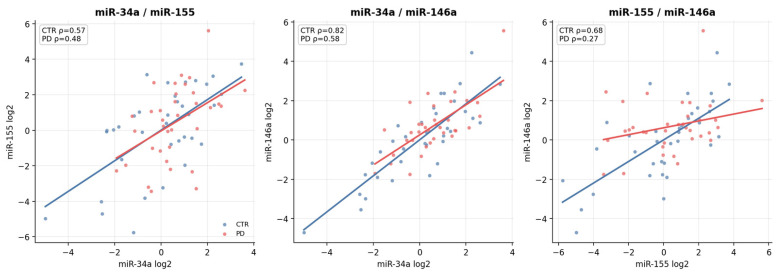
Pairwise co-expression of miR-34a, miR-155 and miR-146a in healthy controls (blue) versus periodontal disease (red). Regression lines are consistently steeper in controls; the miR-155/miR-146a relationship is markedly weaker in disease (Spearman rho = 0.68 vs. 0.27).

In healthy controls, the three miRNAs move together tightly; miR-34a and miR-146a are almost collinear (ρ = 0.82), and miR-155 and miR-146a are strongly linked (ρ = 0.68), as depicted in Figure 5. In periodontal disease, this coupling loosens substantially: the miR-155/miR-146a relationship falls to ρ = 0.27, and the average pairwise coupling across the three miRNAs drops from |ρ| = 0.72 to 0.47. A label-permutation test (20,000 iterations, robust to the small sample and non-normal data) puts the two strongest pair differences at *p* ≈ 0.056–0.059 and the whole-network difference at *p* ≈ 0.072.

**Figure 6:**
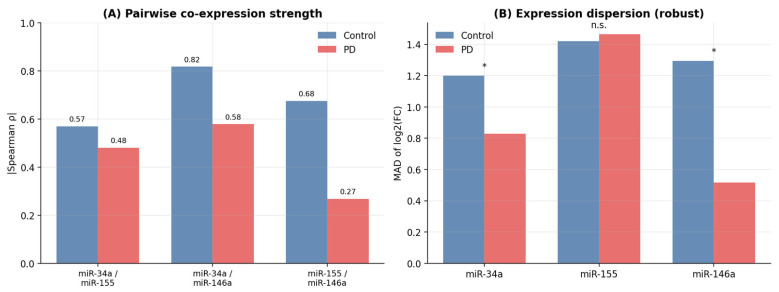
miRNA co-expression coordination and dispersion in health versus disease. (**A**) Absolute pairwise Spearman correlations among the three miRNAs are lower in periodontal disease (PD) patients than in controls for every pair. (**B**) Robust dispersion (median absolute deviation, MAD) of log2 expression is reduced for miR-34a and miR-146a in disease, whereas miR-155 is unchanged (* Levene *p* < 0.05).

Because the three miRNAs did not differ in absolute abundance, we examined whether their coordination differed between groups. In healthy controls, the miRNAs were tightly co-expressed (mean |Spearman rho| = 0.72), whereas, in periodontal disease, this coordination was weaker (mean |rho| = 0.47). The largest change was observed for the miR-155/miR-146a pair (rho = 0.68 in controls versus 0.27 in patients) and the miR-34a/miR-146a pair (0.82 versus 0.58); a 20,000-iteration label-permutation test placed these differences at *p* approximately 0.056–0.059, and the overall correlation structure difference at *p* approximately 0.07 (Figure 6A). In parallel, the expression levels of miR-34a and miR-146a were significantly less dispersed in disease, with the robust median absolute deviation reduced by roughly 30–60% (Levene *p* = 0.033 and 0.024, respectively), whereas miR-155 dispersion was unchanged (Figure 6B). Together, these observations indicate that periodontal disease is accompanied by a partial loss of coordinated miRNA regulation and a compression of expression range, rather than by a shift in mean abundance. Because miR-34a and miR-146a also showed reduced dispersion in disease (Figure 6B) and range restriction mechanically attenuates correlation coefficients, we assessed whether the co-expression change was separable from the variance change. Correcting the disease correlations for their reduced dispersion (Thorndike Case II) returned a miR-34a/miR-146a association close to the control value (0.58 corrected to 0.73, versus 0.82 in controls), indicating that its apparent decline is largely a range restriction effect, whereas the miR-155/miR-146a association remained below the control value after correction (0.27 corrected to 0.38, versus 0.68). Because none of the permutation *p*-values reaches significance (0.056–0.072) and the co-expression and dispersion changes cannot be cleanly disentangled in a sample of this size, we present them explicitly as a single, jointly hypothesis-generating observation, rather than as two independent effects [27,28]. Because only three miRNAs were analyzed, the inferred co-expression network represents a minimal regulatory module, rather than a genome-wide network.

### 3.6. Age as a Confounding Factor

**Figure 7:**
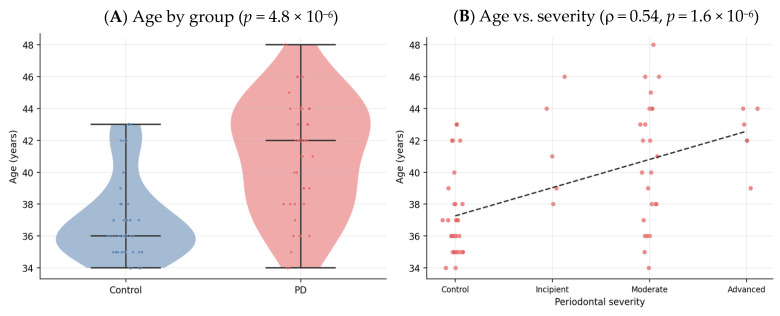
Age as a confounding factor. (**A**) Patients with periodontal disease (PD) are significantly older than controls. (**B**) Age rises with periodontal severity (control -> incipient -> moderate -> advanced). Age alone discriminates the groups better than any miRNA.

Because patients and controls were not age-matched, age was examined as a potential confounder in more detail. Patients were significantly older than controls (41.1 +/− 3.6 versus 37.1 +/− 2.6 years; Mann–Whitney *p* < 0.001; Figure 7A), and age increased with disease stage (Spearman rho = 0.54, *p* < 0.001; Figure 7B). Age is therefore the principal driver of between-group differences in this cohort and a critical confounder for any salivary miRNA comparison; future studies should age-match cases and controls or adjust for age explicitly [26]. The magnitude of this age effect (age-alone AUC = 0.82) is notable for a mean difference of only about four years within the narrow 35–45-year window studied; it is consistent with the strong, monotonic relationship between age and periodontal attachment loss, but it should be interpreted as reflecting age itself, rather than a specific property of the miRNAs, and a recruitment characteristic correlated with age cannot be entirely excluded.

## 4. Discussion

The identification of reliable molecular biomarkers has become a major focus of biomedical research, as they can improve disease diagnosis, prognosis, and therapeutic monitoring. Among these biomarkers, miRNAs have attracted considerable attention due to their ability to regulate gene expression and influence a wide range of biological processes, including inflammation, immunity, cell proliferation, and tissue remodeling [29,30,31]. Altered miRNA expression profiles have been reported in numerous human diseases, including cancer, cardiovascular disorders, autoimmune diseases, and chronic inflammatory conditions, highlighting their potential as diagnostic and prognostic tools [32,33]. In periodontal disease, increasing evidence suggests that specific miRNAs contribute to the regulation of host inflammatory responses and tissue destruction, supporting their potential roles as biomarkers of disease activity and severity [34]. In this context, the present study investigated the expression profiles of miR-34a, miR-155, and miR-146a and their associations with clinical parameters of periodontal disease severity.

MiRNAs have emerged as key epigenetic regulators of periodontal disease, influencing multiple biological processes involved in disease initiation and progression, including immune response modulation, inflammatory signaling, osteoclastogenesis, and tissue remodeling. Dysregulated miRNA expression has been consistently observed in the gingival tissues, saliva, and gingival crevicular fluid of patients with periodontitis, suggesting their potential roles as both pathogenic mediators and biomarkers of disease activity [35,36]. Among the most extensively studied miRNAs in periodontal disease are miR-146a and miR-155, which regulate TLR and NF-κB signaling pathways and are closely associated with inflammatory responses in periodontal tissues [37]. Other miRNAs, including miR-21, miR-223, miR-200 family members, and miR-34a, have also been implicated in the regulation of bone metabolism, immune cell differentiation, and periodontal tissue destruction [34,36]. The growing body of evidence supporting the involvement of these miRNAs highlights their potential utility as diagnostic, prognostic, and therapeutic targets in periodontitis.

MiR-34a has emerged as an important regulator of periodontal disease through its involvement in inflammation, osteoclast differentiation, and alveolar bone remodeling. Previous studies have demonstrated altered expression of miR-34a in periodontal tissues and biological fluids from patients with periodontitis, suggesting its participation in disease progression. Functionally, miR-34a modulates pathways associated with osteoclastogenesis and bone resorption by targeting genes involved in cell differentiation and inflammatory signaling, thereby contributing to periodontal tissue destruction [34]. Furthermore, recent evidence has identified miR-34a-5p among the miRNAs with potential diagnostic value in periodontitis, although its performance appears lower than those of established biomarkers such as miR-146a and miR-155 [38]. Given its dual role in regulating inflammatory responses and bone metabolism, miR-34a may participate in periodontal tissue biology. Importantly, miR-34a is also a recognized effector of cellular senescence and the senescence-associated secretory phenotype, processes that intensify with age and may confound its interpretation in cross-sectional cohorts [18,19].

Our results also differ from the meta-analysis by Memon et al. [38], but in the direction opposite to our original interpretation: whereas that work reported modest diagnostic value for miR-34a-5p relative to miR-146a and miR-155, we did not detect any significant differential abundance for salivary miR-34a. Such discrepancies are common in salivary miRNA research and likely reflect differences in sample type (saliva versus serum or gingival biopsy), profiling platform and normalization strategy, cohort age structure, and the specific chronic phenotype studied. They underscore the importance of rigorous reference gene selection and normalization for salivary miRNA quantification [25], and the value of reporting negative as well as positive findings to counter publication bias in biomarker discovery.

In the present cohort, contrary to our initial hypothesis and to some earlier reports, miR-34a was not differentially expressed between periodontitis patients and controls, showed no robust correlation with clinical attachment loss, and did not decrease progressively across disease stages. The apparent group separation seen in preliminary analyses was traced to the age imbalance between cohorts, rather than to miR-34a itself. These null findings do not exclude a biological role for miR-34a in periodontal tissue—where it participates in osteoclast differentiation, inflammatory signaling and senescence [17,18,19,34]—but they indicate that its salivary abundance is not a useful stand-alone diagnostic or severity marker in middle-aged patients. A notable secondary observation was that miR-34a was positively co-expressed with miR-146a (rho = 0.73) and miR-155 (rho = 0.53) across the cohort, and that this co-expression was weaker in disease, pointing to a possible network-level, rather than abundance-level, alteration.

Unlike miR-34a, miR-155 did not exhibit significant differential expression between periodontitis patients and healthy controls in the present study, nor was it associated with clinical attachment loss or disease severity. Although miR-155 has been widely recognized as a pro-inflammatory miRNA involved in macrophage activation, cytokine production, and periodontal inflammation, our findings suggest that its expression remained relatively stable across the analyzed cohort. Similar variability has been reported in the literature, where miR-155 expression appears to be influenced by disease phenotype, inflammatory burden, and the biological sample analyzed [37]. Nevertheless, a significant positive correlation was identified between miR-155 and miR-146a expression, indicating a potential co-regulation of these transcripts within shared inflammatory signaling pathways. This observation is biologically plausible, as both miR-155 and miR-146a are induced by Toll-like receptor activation and participate in the regulation of NF-κB-mediated immune responses through a well-characterized feedback module, albeit with partially opposing functions [20,39,40]. Notably, this co-regulation was more pronounced in healthy controls than in patients in our data, consistent with the idea that the miR-146a/miR-155 axis becomes dysregulated during chronic periodontal inflammation. Therefore, while miR-155 did not demonstrate diagnostic or prognostic value in the present cohort, its association with miR-146a may reflect its involvement in the broader regulatory network governing periodontal inflammation.

Overall, the present study did not identify any salivary miRNA that discriminates periodontal disease on the basis of abundance. Instead, the two reproducible signals were the confounding effect of age and an exploratory, network-level reduction in miRNA co-expression and dispersion in disease. These observations reframe the potential values of miR-34a, miR-155 and miR-146a in saliva from single-marker abundance toward age-adjusted, co-expression-based approaches that remain to be validated.

## 5. Conclusions

The present study did not confirm salivary miR-34a, miR-155 or miR-146a as differentially expressed markers of periodontal disease. None of the three miRNAs differed significantly between patients and controls, none correlated with clinical attachment loss after correction, and miR-34a showed no stand-alone diagnostic value (AUC = 0.58). The strongest determinant of group membership was patient age, which must be regarded as a key confounder in salivary miRNA biomarker studies.

The three miRNAs were, however, positively co-expressed, and this coordination was attenuated in disease in an exploratory analysis. This suggests that periodontal disease may be associated with altered miRNA co-regulation, rather than with a change in the absolute level of any single miRNA—a hypothesis that abundance-based analyses cannot capture.

Taken together, these findings argue for caution in interpreting single salivary miRNAs as periodontal biomarkers; for explicit age matching or adjustment in future studies; and for larger, age-matched cohorts to test whether miRNA co-expression patterns hold promise as network-level indicators of periodontal disease. Reporting these predominantly negative results is intended to help counter publication bias and to guide the design of future salivary miRNA investigations.

## 6. Study Limitations

Several limitations should be acknowledged. First, the sample was modest (35 patients, 35 controls) and the isolated severity subgroups were very small (incipient n = 5, advanced n = 6), leaving stage-specific comparisons underpowered; the corresponding analyses are therefore exploratory. Second, patients and controls were not age-matched: patients were four years older on average, and because age is strongly associated with periodontal destruction, it acts as a confounder that limits the interpretation of any between-group comparison. Third, the cross-sectional design precludes causal inference. Fourth, participants were restricted to individuals aged 35–45 years without major systemic comorbidities, limiting generalizability. Fifth, expression was assessed only in saliva and only for three miRNAs, so the findings may not reflect molecular changes within gingival tissue or the broader miRNA network. Sixth, salivary miRNA normalization is intrinsically challenging; although RNU48 and U6 Ct values were stable between groups, we cannot exclude residual normalization variance, and future work should validate reference genes using dedicated stability algorithms [25]. Finally, the exploratory differential co-expression signal was of borderline statistical significance and may be influenced by range restriction; it requires confirmation in a larger, independent, age-matched validation cohort before any clinical inference can be drawn. In addition, a post hoc sensitivity analysis indicated that, with 35 participants per group, the study had 80% power (α = 0.05, two-sided) to detect a standardized between-group difference of Cohen’s d ≈ 0.68, corresponding to approximately a two-fold change in miR-34a expression; the observed effects were considerably smaller (|d| ≈ 0.2–0.4). The present negative result therefore excludes moderate-to-large differences, but cannot exclude a small true effect.

## Figures and Tables

**Table 1:** Endogenous controls (RNU48 and U6) and the target miRNAs (hsa-miR-34a, hsa-miR-146a, and ipu-miR-155) included in the qRT-PCR analysis, together with their nucleotide sequences.

No.	miRNAs	Sequence	Assay ID
1	RNU48	GATGACCCCAGGTAACTCTGAGTGTGTCGCTGATGCCATCACCGCAGCGCTCTGACC	001006
2	U6	GTGCTCGCTTCGGCAGCACATATACTAAAATTGGAACGATACAGAGAAGATTAGCATGGCCCCTGCGCAAGGATGACACGCAAATTCGTGAAGCGTTCCATATTTT	001973
3	hsa-miR 34a	UGGCAGUGUCUUAGCUGGUUGU	000426
4	hsa-miR 146a	UGAGAACUGAAUUCCAUGGGUU	000468
5	ipu-miR-155	UUAAUGCUAAUCGUGAUAGGGGUU	467534_mat

**Table 2:** Demographic and clinical data of the study cohort.

Parameter	Control (n = 35)	Periodontal Disease (n = 35)
Age (Mean ± SD)	37.1 ± 2.6	41.1 ± 3.6
Gender (Female/Male)	18/17	20/15
Severity (Incipient/Moderate/Advanced)	-	5/24/6

**Table 3:** Differential expression analysis of miRNAs in periodontal disease and healthy controls.

miRNA	Control Median	PD Median	*p*-Value	Adjusted *p*-Value (FDR)	Interpretation
miR-34a	0.29	0.46	0.28	0.42	Not significant
miR-155	0.19	0.79	0.66	0.66	Not significant
miR-146a	0.16	0.51	0.13	0.40	Not significant

## Data Availability

The data presented in this study is available on request from the corresponding author due to ethical restrictions.

## Supplementary Materials

The following supporting information can be downloaded at: https://www.mdpi.com/article/10.3390/cells15151392/s1, Table S1: data set.
